# Quantifying impact loading for osteoporosis prevention: a study on the relationship between ground reaction forces and wearable impact data

**DOI:** 10.1007/s11657-026-01715-8

**Published:** 2026-09-03

**Authors:** Gonzalo Reverte-Pagola, Horacio Sánchez-Trigo, Carlos Rangel, Sergio Tejero, Borja Sañudo

**Affiliations:** 1 Department of Physical Education and Sport, University of Seville, Seville, Spain; 2 School of Humanities, Education and Sport, Universidad CEU Fernando III, CEU Universities, Bormujos, Sevilla Spain; 3 Orthopaedic Department of University Hospital Virgen del Rocío, Head of Foot and Ankle Unit, Department of Surgery, University of Seville, Seville, Spain

**Keywords:** Wearable sensors, Osteogenic loading, Osteoporosis prevention, Accelerometry, Clinical exercise prescription

## Abstract

**Summary:**

High-impact exercise can strengthen bones, but clinical use is limited because mechanical loading is difficult to measure outside laboratories. We validated a wrist-worn accelerometer against force-plate ground reaction forces and found that wearable data reliably reflect impact loading. This approach may enable practical monitoring of bone-stimulating exercise in real-world settings.

**Purpose:**

Mechanical loading must surpass a minimum intensity threshold to stimulate osteogenic adaptation, yet clinicians lack practical tools to quantify impact dose outside laboratory settings. Wearable accelerometers could enable safe, scalable monitoring of bone-strengthening exercise, but their validity for estimating osteogenic loading remains uncertain. This study examined whether wrist-worn accelerometry can support approximate estimation and coarse classification of platform-derived impact loading during maximal countermovement jumps in postmenopausal women.

**Methods:**

Thirty-eight women were enrolled; 37 contributed 125 valid CMJs with concurrent force-platform and wrist-accelerometer recordings. The primary model predicted peak platform-derived vertical acceleration from peak wrist vector-magnitude acceleration and body mass. Coefficient inference used linear regression with participant-clustered robust standard errors. Predictive performance and four-class osteogenic classification (< 2 g, 2– < 3 g, 3– < 3.9 g, ≥ 3.9 g) were evaluated using leave-one-subject-out cross-validation. Fuller anthropometric models and residual-based outlier filtering were examined as sensitivity analyses.

**Results:**

In the primary reduced model, wrist vector magnitude and body mass were both independently associated with peak platform acceleration. In leave-one-subject-out validation, the model explained 44.3% of the variance (*R*^2^ = 0.443), with MAE = 5.83 and RMSE = 7.99 m·s⁻^2^. Mean bias was 0.05 m·s⁻^2^, with 95% limits of agreement from − 15.66 to 15.77 m·s⁻^2^. Four-class classification accuracy was 0.544, with quadratic weighted *κ* = 0.639; misclassifications occurred predominantly between adjacent bins. The fuller anthropometric model did not improve out-of-sample performance.

**Conclusions:**

Wrist-worn accelerometry provided limited precision for per-jump estimation of platform-derived loading, even under constrained laboratory conditions. However, it retained moderate utility for coarse, bin-based classification of impact intensity during maximal CMJs performed with hands on hips. Its most defensible application is session-level monitoring and conservative progression in home- and community-based osteogenic exercise programs, rather than replacement of laboratory force-platform measurement.

**Supplementary Information:**

The online version contains supplementary material available at https://doi.org/10.1007/s11657-026-01715-8.

## Introduction

Osteoporosis is a major public health concern, characterized by the deterioration of bone tissue microarchitecture and low bone mineral density (BMD), leading to reduced bone strength and an increased risk of fractures [1]. It is estimated that more than 200 million people worldwide suffer from osteoporosis, and this number continues to rise due to population aging and lifestyle changes [2]. Osteoporosis imposes a significant economic burden on healthcare systems worldwide. In the USA, annual fracture-related costs were estimated at $48.8 billion in direct medical costs in 2018 and are projected to rise to $81.5 billion by 2040; when indirect societal costs (e.g., productivity losses and informal caregiving) are included, total annual costs increase to $57.0 billion and $95.2 billion, respectively [3]. Similarly, in the European Union, the total direct costs of osteoporotic fractures and their management amount to approximately €57 billion each year [4]. These substantial costs underscore the critical need for effective prevention and management strategies to mitigate the financial impact of osteoporosis on global healthcare systems [5]. Non-pharmacological approaches, including dietary modifications and physical exercise, are key strategies for managing osteoporosis. Importantly, current consensus recommendations indicate that appropriately prescribed physical activity, including impact and resistance-based exercise, is generally safe and well tolerated in individuals with osteoporosis. When exercise programs are tailored to individual capacity and progressed gradually, potential benefits can be achieved without increasing the risk of adverse events [6]. Strong epidemiological and interventional evidence underscores the critical role of physical activity in bone health, showing that individuals who engage in regular exercise experience a significantly lower risk of fractures [6].

Bone responds to mechanical loading by adapting its structure to better resist external forces, a process known as bone modeling. Consequently, activities that generate higher intensity or higher strain rates promote osteogenic signaling and osteoblast differentiation, enhancing the production of healthy bone cells [7]. For exercise to stimulate bone adaptation, mechanical loading must exceed the habitual strain range in the predominant loading direction, requiring sufficient intensity, frequency, and variability [8]. Consequently, research suggests that high-impact loads are most effective in improving bone health. High-impact activities such as running and jumping provide superior osteogenic benefits compared to low-intensity alternatives [9]. Although high-impact loading is a potent osteogenic stimulus, high-load resistance exercise has also demonstrated efficacy in promoting bone adaptation, particularly through muscle-mediated skeletal loading [10]. However, among postmenopausal women, the evidence on the effectiveness of jumping programs for enhancing bone mass remains inconclusive [11]. While a meta-analysis [12] reported significant increases in premenopausal women in hip BMD following short-duration (< 30 min) high-impact exercise protocols (e.g., vertical jumping), other meta-analyses suggest that impact-only interventions (e.g., jumping, skipping) primarily benefit femoral neck BMD, whereas combined impact-resistance training (e.g., circuit training, group fitness) produces significant effects at multiple skeletal sites [13]. Similarly, Florence et al. [14] found that jump training significantly increased femoral neck BMD but had no measurable effects on the total hip or trochanter. Kistler-Fischbacher et al. [13] also reported no statistically significant effects of impact-only training on lumbar spine, total hip, or femoral neck BMD. However, they suggested that certain intensities (i.e., ground reaction forces (GRFs) exceeding two times body weight (BW)) may be required to elicit an osteogenic response and that the femoral neck is more responsive to impact exercises than other skeletal sites.

The optimal exercise dose for bone maintenance, encompassing frequency, intensity, duration, and type, remains unclear [15–17] and the optimal force that enhances bone formation is still unknown [9]. The threshold varies between individuals, and even between bone sites, according to physical activity habits and other influencing factors [18]. Intensity appears to be the most determinant parameter influencing these effects. A recent meta-analysis by Kistler-Fischbacher et al. [13] established a precise classification of the programmed intensity across different studies. Authors suggested that GRFs of at least twice BW may be necessary to elicit an osteogenic response. However, GRF values exceeding four times BW generate higher magnitude bone-loading strains, which are more effective in promoting bone accretion [19]. Therefore, low-intensity activities (< 2BW), such as walking, jogging, or cycling, appear insufficient to elicit a significant bone response, whereas high-intensity loading (> 4BW), including jumps with stiff-legged landings, may provide an adequate osteogenic stimulus [11]. It should be noted that evidence supporting higher impact thresholds (> 4 × BW) includes data from adolescent populations [19], and that randomized controlled trials applying these loads in postmenopausal women remain limited and heterogeneous.

While progressive exposure to impact loading, starting with 10 jumps per day and increasing to 60, at intensities exceeding 4BW, performed at least four times per week, has been shown to preserve BMD in postmenopausal women [6, 11, 14, 19–23], not all studies have reported positive outcomes. For instance, Bassey et al. (1998) investigated the effects of a high-impact exercise intervention involving 50 vertical jumps per session, 6 days per week, with a mean jump height of 8.5 cm and GRF of approximately 3BW [24]. In this randomized controlled trial, significant increases in femoral BMD were observed in premenopausal women, whereas no significant changes were detected in postmenopausal women, despite higher absolute ground reaction forces in the latter group. These findings suggest that the applied loading stimulus may have been insufficient to elicit measurable osteogenic adaptations in postmenopausal women. Similarly, Marin-Cascales et al. (2019) examined the effects of a combined impact training and walking program in postmenopausal women, consisting of 4–6 sets of 10 vertical jumps (ranging from 5 to 25 cm in height) alongside 30–60 min of walking, performed three times per week over 24 weeks [25]. Despite progressive loading and high adherence, no statistically significant increases in femoral neck bone mass were observed. Lastly, Altai et al. [9] suggested that countermovement jumps (CMJs) exercises have a limited effect on bone health, even when performed with maximal effort, particularly in the hip and ankle joint regions.

These discrepancies underscore the complexity of designing effective impact-loading interventions and suggest that specific loading characteristics are critical in determining their osteogenic effectiveness. However, uncertainty persists regarding the optimal loading patterns required for bone adaptation, as skeletal responsiveness to mechanical stimuli is strongly influenced by biological and nutritional factors, including calcium availability, across the lifespan [26]. To predict the osteogenic impact of various exercises, quantitative data on the forces exerted on the joints of the lower limbs are required [9]. Given the necessity of reaching a minimum threshold of mechanical stimulation for bone adaptation, precise quantification of musculoskeletal loading using wearable activity monitors could be an effective strategy [27]. While international guidelines and recommendations use variables such as BW to determine exercise intensity, individuals often lack access to technologies that provide real-time feedback on the appropriate exercise dose. Therefore, tools that enable more precise control of exercise intensity are needed. These devices could be used in various settings beyond laboratory environments.

Importantly, these mixed findings do not negate the relevance of impact loading per se, but rather highlight the need for accurate quantification of loading intensity to ensure that exercise prescriptions reach osteogenic thresholds safely and consistently. Accordingly, this study aims to evaluate the accuracy of a commercially available wrist-worn accelerometer for estimating ground reaction-based impact-loading intensity in laboratory settings. Specifically, we examine the relationship between peak wrist acceleration recorded by the wearable device and peak vertical ground reaction forces (VGRFs) obtained from a laboratory-grade force platform. Establishing this association will help assess whether wearable accelerometry can serve as a practical and valid proxy for monitoring osteogenic loading outside controlled environments, thereby facilitating the real-world application of evidence-based intensity thresholds for osteoporosis-preventive exercise.

## Methods

### Study design

This laboratory-based, cross-sectional validation study was conducted under controlled conditions to evaluate the association between measurements by a wrist-worn accelerometer and by a force platform during CMJs. Participants performed repeated CMJs on an instrumented force platform while wrist acceleration was recorded concurrently. The primary platform-derived outcome was peak vertical acceleration (m·s⁻^2^), calculated from the VGRFs. The wearable predictor was peak wrist acceleration vector magnitude. The study protocol was approved by the Andalusian Biomedical Research Ethics Committee (approval code: 0486-N-22) and conducted in accordance with the Declaration of Helsinki. Written informed consent was obtained from all participants prior to enrollment.

### Participants

Participants were recruited from the gynecology units of Virgen del Rocío University Hospital (Seville, Spain) and through public outreach via media advertisements and informational brochures in primary care centers. Eligibility criteria included women aged > 40 years and ≤ 10 years postmenopause, performing < 150 min/week of moderate-to-vigorous physical activity, and able to safely execute brief high-impact jumps in a supervised laboratory setting, as determined through medical screening and a supervised familiarization phase involving practice jumps without pain, discomfort, or instability. Exclusion criteria comprised surgically induced menopause, active cancer treatment, body mass index < 18 kg·m⁻^2^, current smoking, excessive alcohol consumption, recent hormone replacement therapy (< 3 months), recent upper or lower limb fracture, or any musculoskeletal condition likely to compromise safe participation. The planned sample size was 40 participants; the final analyzed sample included all volunteers with complete and valid wrist-accelerometry and force-platform recordings for at least one CMJ, as detailed in the “Results” section.

### Experimental procedure

Testing occurred in a biomechanics laboratory under supervised conditions. On the day of testing, participants completed a standardized warm-up consisting of light aerobic activity and task rehearsal. Anthropometric measurements (body mass, height, waist, and hip circumferences) were obtained in duplicate and the mean of each pair was recorded. A wrist-worn accelerometer was secured to the participant’s non-dominant wrist according to the manufacturer’s instructions, ensuring a snug fit and unrestricted movement. The device recorded wrist motion continuously throughout the tasks.

Participants performed maximal CMJs on a force platform, starting from an upright standing position with feet shoulder-width apart. They executed a rapid downward movement followed immediately by a maximal vertical jump. Hands were kept on the hips to avoid arm-swing contribution and standardize the movement. Verbal encouragement was provided to promote consistent maximal effort. At least two familiarization jumps were performed prior to data collection. Three trials were obtained per participant, separated by brief rest periods (~ 1 min) to minimize fatigue. Trials with technical artifacts (e.g., loss of balance, incomplete foot contact, premature interruption) were repeated. If a trial was invalid due to technical or execution-related issues, additional attempts were performed, resulting in a variable number of total attempts (typically 3–6) per participant. Safety was prioritized, and the protocol was discontinued if participants experienced pain, dizziness, or discomfort. The number of valid trials per participant and total analyzed trials are reported in the “Results” section.

### Instrumentation

VGRFs were recorded using a laboratory-grade force platform (Kistler 9286BA, Winterthur, Switzerland) operating at 1000 Hz. Only the vertical channel (Fz) was used for analysis. The platform was zeroed before each session and calibrated according to the manufacturer’s procedures, following routine laboratory quality-control checks.

Wrist acceleration was captured with a Fitbit Versa 3 smartwatch (Fitbit Inc., San Francisco, CA, USA) worn on the non-dominant wrist. The device contains a tri-axial Micro-Electro-Mechanical Systems accelerometer and, in this protocol, produced raw x, y, and z signals at a nominal sampling frequency of ~ 50 Hz. The strap was adjusted to ensure a secure fit and unobstructed movement. Data were exported in raw form for offline processing.

Anthropometric variables included body mass, height, and waist and hip circumferences. All measurements were obtained in duplicate using standard procedures with a digital scale, a wall-mounted stadiometer, and a flexible, non-elastic measuring tape. The mean of each duplicate pair was used in subsequent analyses.

### Data handling and preprocessing

Force-plate signals were imported as time series of the Fz sampled at 1000 Hz. For each trial, BW force was computed as mass × 9.8 m·s⁻^2^, and net vertical force was obtained by subtracting this value from Fz. Net force was numerically integrated over time (Δ*t* = 0.001 s) to estimate vertical velocity, and vertical acceleration was then derived by first-order finite differencing of the velocity trace. Flight was identified using a conservative threshold of Fz < 30 N. No filtering or smoothing was applied to the force, velocity, or acceleration signals. This approach was chosen to preserve peak signal integrity; however, it may have increased trial-level variability and should be addressed in future studies. The platform-derived acceleration is reported in m·s⁻^2^.

Wrist accelerometry was parsed as raw tri-axial components (x, y, z) from a device operating at a nominal sampling frequency of ~ 50 Hz. The vector magnitude was computed as √(× 2 + y2 + z2). For each analyzed CMJ, the peak vector magnitude was identified, and the corresponding x, y, and z values at that instant were recorded. Wrist accelerations were retained in the device’s native units (m·s⁻^2^) without gravity removal or unit conversion. Wrist and force-platform data were paired using subject and trial identifiers, and analysis windows were manually defined from the force–time trace by the same analyst to ensure consistent identification of complete jump phases. Only windows containing a complete, biomechanically valid jump, characterized by clear take-off, flight, and landing phases, were included in the analysis.

The analytic dataset was constructed by merging trial-level features with anthropometric measurements (body mass, height, waist and hip circumferences) using a subject code join.

### Outcomes

The primary outcome was the per-trial association between the peak wrist accelerometry vector magnitude and the peak vertical acceleration measured by the force platform. Peak platform acceleration was expressed in m·s⁻2 and derived from the time-differentiated velocity trace, while wrist acceleration was retained in the device’s native units and summarized as the maximum vector magnitude within each analysis window.

Secondary outcomes included agreement between observed and cross-validated predicted platform accelerations and four-class classification of impact intensity. Classification thresholds were defined at 2 g, 3 g, and 3.9 g, corresponding to 19.62, 29.43, and 38.259 m·s⁻^2^, respectively. These cut-offs yielded four ordered classes: < 2 g, 2– < 3 g, 3– < 3.9 g, and ≥ 3.9 g. The 3.9 g cut-off was selected because Vainionpää’s work identified impacts above this level as osteogenic, showing clear associations with positive bone-loading responses [27]. Classification performance was evaluated by comparing threshold-based labels derived from the model-estimated platform acceleration against labels derived from the force-plate signal.

### Statistical analysis

Statistical analyses were conducted in Python (version 3.10) using the pandas, NumPy, scikit-learn, statsmodels, and Matplotlib libraries. Participant characteristics and trial counts were summarized using means and standard deviations for continuous variables and frequencies for counts. Because multiple CMJs were contributed by the same participant, all inferential analyses explicitly accounted for within-subject clustering.

The primary regression model predicted trial-level peak platform-derived vertical acceleration (m·s⁻^2^) from peak wrist vector-magnitude acceleration and body mass. This reduced specification was adopted as the primary model because it provided a more parsimonious calibration framework and generalized better than the fuller anthropometric specification while limiting overfitting and collinearity in a dataset with repeated observations per participant. Regression coefficients, two-sided 95% confidence intervals, and p-values were estimated using ordinary least squares models with cluster-robust standard errors grouped by participant. Standardized coefficients were additionally calculated to aid interpretation of the relative contribution of each predictor.

Predictive performance was evaluated out of sample using leave-one-subject-out cross-validation (LOSO-CV). In each fold, all trials from one participant were held out, the model was fitted using the remaining participants, and predictions were generated for the held-out subject. Cross-validated performance was summarized using the coefficient of determination (*R*^2^), mean absolute error (MAE), root mean squared error (RMSE), mean bias, and 95% limits of agreement. Agreement between observed and LOSO-predicted platform accelerations was further examined using paired t-tests and Bland–Altman analysis, with limits of agreement calculated as bias ± 1.96 × SD of the differences.

Four-class classification was assessed by discretizing both observed and LOSO-predicted platform accelerations using thresholds at 2 g, 3 g, and 3.9 g, converted to SI units as 19.62, 29.43, and 38.259 m·s⁻^2^, respectively. This yielded four ordered classes: < 2 g, 2– < 3 g, 3– < 3.9 g, and ≥ 3.9 g. Values exactly equal to a threshold were assigned to the higher category. Classification performance was summarized using confusion matrices, class-wise precision, recall, and F1-scores, overall accuracy, quadratic weighted Cohen’s *κ*, and mean absolute error in class index.

Two sensitivity analyses were conducted. First, a fuller anthropometric model including body mass, height, waist circumference, hip circumference, and wrist vector magnitude was evaluated using the same clustered inference and LOSO-CV procedure. Second, an exploratory residual-based outlier filter was applied using an interquartile-range rule, after which both the reduced and fuller models were re-estimated. These filtered analyses were treated as sensitivity analyses rather than primary results.

All tests were two-sided, and statistical significance was set at *α* = 0.05.

## Results

### Participant flow and characteristics

A total of 40 postmenopausal women were screened for eligibility. Two were excluded prior to enrolment because they did not meet the inclusion criteria. The remaining 38 participants attended the laboratory session and completed anthropometric assessment and jump testing. After exclusion of technically unusable recordings, including mislabelled files that prevented accurate pairing of force-platform and wrist-accelerometry signals and trials affected by force-platform calibration problems, the primary analytic dataset comprised 125 valid countermovement jumps from 37 participants.

As a sensitivity analysis, an exploratory residual-based interquartile-range filter identified four outlying trials. This yielded a filtered dataset of 121 jumps from 36 participants, which was used only for secondary analyses and not as the primary basis for inference. Accordingly, all main regression, agreement, and classification results are presented for the unfiltered dataset, whereas filtered analyses are reported as sensitivity analyses. Participant characteristics of the enrolled sample are summarized in Table 1.

**Table 1:** Participant characteristics of the enrolled sample (*n* = 38)

Variable	Mean ± SD
Age (years)	57.5 ± 4.1
Height (cm)	162.3 ± 4.75
Body mass (kg)	71.2 ± 18.2
BMI (kg·m⁻^2^)	27.0 ± 6.5
Waist circumference (cm)	86.0 ± 14.1
Hip circumference (cm)	107.0 ± 14.6

*Note:* One participant contributed anthropometric data but no usable paired force-platform and wrist-accelerometry recordings

### Regression performance and agreement

In the primary reduced model, trial-level peak platform-derived vertical acceleration (m·s⁻^2^) was predicted from peak wrist vector-magnitude acceleration and body mass. Both predictors contributed independently to model performance in participant-clustered regression, with wrist vector magnitude showing the strongest association with the criterion measure and body mass providing a smaller but meaningful additional contribution. Detailed coefficient estimates, 95% confidence intervals, *p*-values, and standardized coefficients are presented in Table 2.

**Table 2:** Primary reduced model predicting peak platform-derived vertical acceleration from wrist vector magnitude and body mass (125 trials from 37 participants)

Panel A. Clustered regression coefficients				
Predictor	β	95% CI	p-value	Standardized β
Intercept	9.82	0.34 to 19.29	0.042	—
Body mass	− 0.188	− 0.259 to − 0.117	< 0.001	− 0.305
Wrist vector magnitude	0.748	0.546 to 0.950	< 0.001	0.600
Panel B. Leave-one-subject-out predictive performance and agreement				
Metric		Value		
Observations, *n*		125		
Participants, *n*		37		
*R*^2^		0.443		
MAE (m·s⁻^2^)		5.83		
RMSE (m·s⁻^2^)		7.99		
Mean bias (m·s⁻^2^)		0.05		
95% limits of agreement (m·s⁻^2^)		− 15.66 to 15.77		
Paired *t*-test		*t*(124) = 0.07, *p* = 0.943		

Abbreviations: CI, confidence interval; MAE, mean absolute error; RMSE, root mean squared error.

Note: Coe icients were estimated using ordinary least squares with participantclustered robust standard errors. Predictive performance and agreement were evaluated using leave-one-subject-out cross-validation. Standardized coe icients are provided to facilitate comparison of predictor importance across variables measured on di erent scales.

**Fig. 1:**
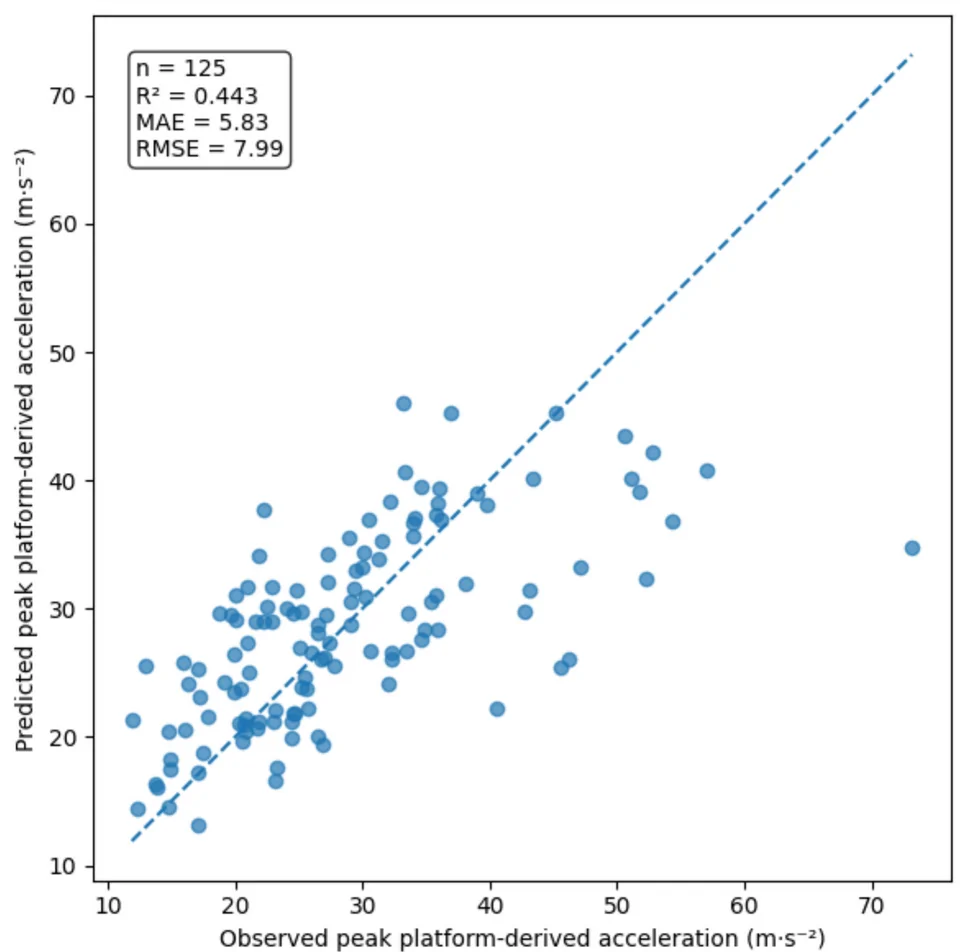
Observed versus leave-one-subject-out predicted peak platform-derived vertical acceleration for the primary reduced model

When predictive performance was evaluated out of sample using leave-one-subject-out cross-validation, the reduced model explained 44.3% of the variance in held-out observations (*R*^2^ = 0.443), with MAE = 5.83 m·s⁻^2^ and RMSE = 7.99 m·s⁻^2^. Observed versus cross-validated predicted values are shown in Fig. 1.

The fuller anthropometric model did not improve out-of-sample performance relative to the reduced model (LOSO *R*^2^ = 0.385 vs 0.443; accuracy = 0.480 vs 0.544 in the unfiltered dataset) and is therefore reported only as a sensitivity analysis in Supplementary Table S1; clustered coefficient estimates for the fuller anthropometric model are provided in Supplementary Table S2.

**Fig. 2:**
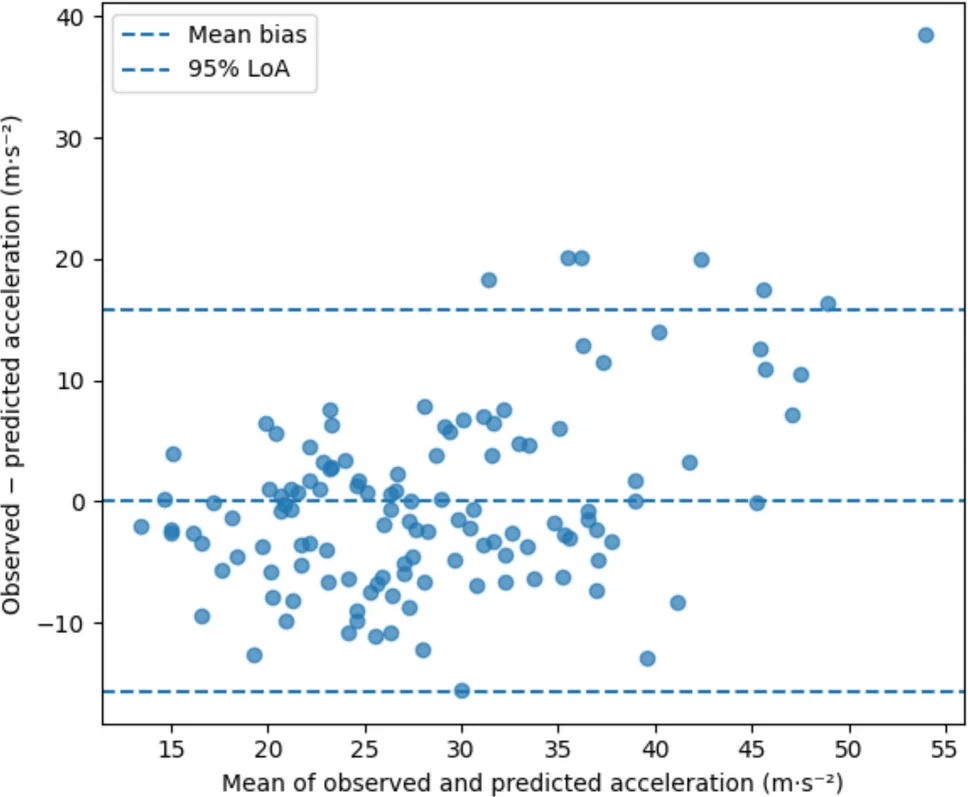
Bland–Altman plot comparing observed and leave-one-subject-out predicted peak platform-derived vertical acceleration for the primary reduced model

Agreement between observed and LOSO-predicted platform accelerations showed minimal mean bias (0.05 m·s⁻^2^), but relatively wide 95% limits of agreement (− 15.66 to 15.77 m·s⁻^2^), indicating limited precision at the individual-jump level. There was no evidence of a systematic mean difference between observed and cross-validated predicted values (paired *t*(124) = 0.07, *p* = 0.943). The Bland–Altman plot is presented in Fig. 2.

For completeness, exploratory residual-based outlier filtering modestly improved the performance of the reduced model in sensitivity analyses (LOSO *R*^2^ = 0.526; MAE = 5.08 m·s⁻^2^; RMSE = 6.58 m·s⁻^2^), but did not materially alter the overall interpretation that wrist accelerometry is better suited to coarse classification than to precise per-jump estimation. Observed versus predicted values and Bland–Altman agreement for the filtered reduced model are shown in Supplementary Figures S1 and S3, respectively.

### Classification performance

Using leave-one-subject-out predictions from the primary reduced model, observed and predicted platform-derived peak accelerations were discretized into four osteogenic intensity bins using thresholds at 2 g, 3 g, and 3.9 g (19.62, 29.43, and 38.259 m·s⁻^2^, respectively). This yielded the ordered classes < 2 g, 2– < 3 g, 3– < 3.9 g, and ≥ 3.9 g.

In the primary analysis, overall four-class classification accuracy was 0.544, with quadratic weighted Cohen’s *κ* = 0.639, indicating moderate ordinal agreement between observed and predicted impact categories. The mean absolute error in class index was 0.488, suggesting that most classification errors were small in magnitude. Class-wise performance is shown in Table 3.

**Table 3:** Four-class classification performance of the primary reduced model using leave-one-subject-out predictions (125 trials from 37 participants)

Class	Precision	Recall	F1-score	Support
< 2 g	0.692	0.450	0.546	20
2– < 3 g	0.625	0.614	0.620	57
3– < 3.9 g	0.381	0.533	0.444	30
≥ 3.9 g	0.571	0.444	0.500	18

**Fig. 3:**
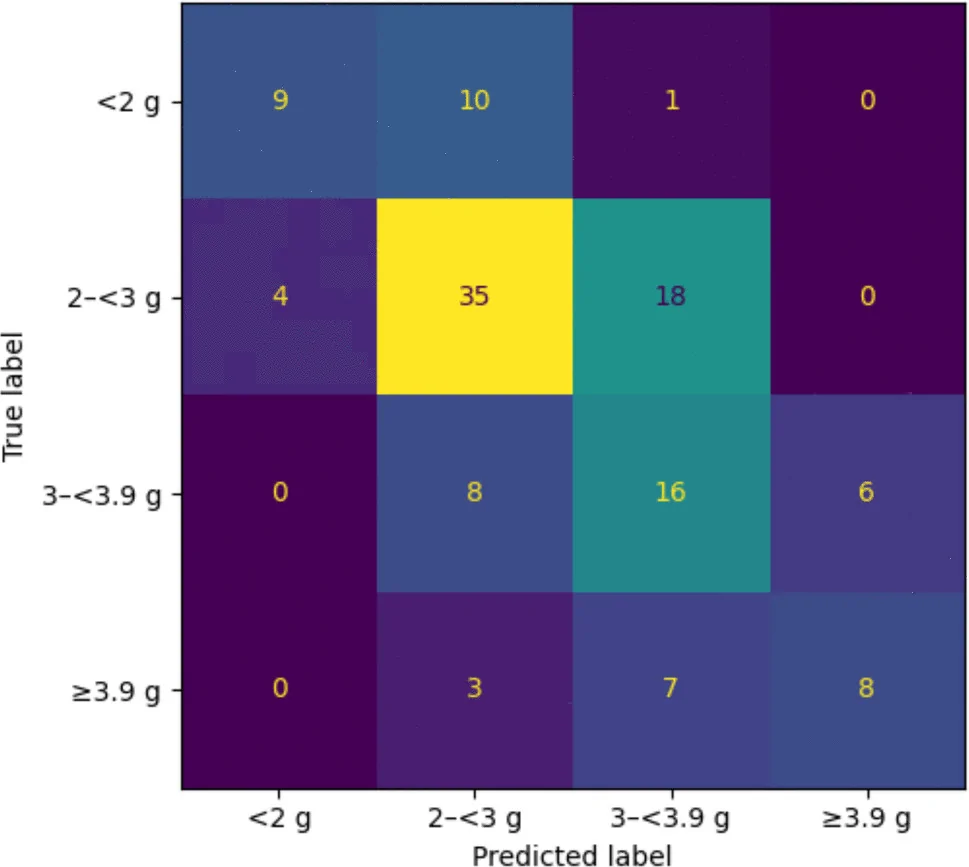
Confusion matrix for four-class osteogenic intensity classification using leave-one-subject-out predictions from the primary reduced model

Most misclassifications occurred between adjacent categories rather than between extreme loading bins, consistent with coarse ordinal discrimination rather than precise threshold classification. The corresponding confusion matrix is shown in Fig. 3.

In sensitivity analyses, residual-based outlier filtering modestly improved classification performance of the reduced model, yielding an accuracy of 0.595 and quadratic weighted *κ* = 0.674, without materially changing the overall interpretation. These filtered results are reported in Supplementary Table S1, and the corresponding confusion matrix is shown in Supplementary Figure S2.

## Discussion

The present study examined whether wrist-worn accelerometry could provide a practical estimate of platform-derived impact loading during maximal countermovement jumps in postmenopausal women. After accounting for repeated measurements and evaluating performance out of sample, the main finding was that wrist accelerometry combined with body mass showed a moderate relationship with peak platform-derived vertical acceleration, but only limited precision for individual-jump estimation. In leave-one-subject-out validation, the primary reduced model explained 44.3% of the variance in held-out observations, with an MAE of 5.83 m·s⁻^2^ and an RMSE of 7.99 m·s⁻^2^. Although mean bias was minimal, the limits of agreement were relatively wide, indicating that wrist accelerometry should not be interpreted as a precise substitute for laboratory force-platform assessment on a jump-by-jump basis.

An important additional finding was that the reduced model, including only wrist vector magnitude and body mass, outperformed the fuller anthropometric specification in out-of-sample prediction and was therefore retained as the primary calibration model. Height, waist circumference, and hip circumference added little incremental value and did not improve generalization. This supports the use of simpler calibration models in future work and reinforces concerns about overfitting when multiple correlated anthropometric variables are included in relatively small repeated-measures datasets. Wrist vector magnitude was the dominant predictor, whereas body mass provided a smaller but still meaningful independent contribution. This pattern is biomechanically plausible, as peripheral acceleration should reflect impact intensity only imperfectly and may benefit from simple body-size calibration. Because unstandardized coefficients are scale-dependent, standardized effects are more informative for comparing predictor importance; in the present study, these confirmed wrist vector magnitude as the strongest contributor while body mass still made a meaningful adjustment.

The classification results suggest that the most defensible role of wrist accelerometry in this context is coarse bin-based classification rather than continuous estimation. Using participant-held-out predictions, four-class classification accuracy was 0.544, whereas quadratic weighted Cohen’s *κ* reached 0.639, indicating moderate ordinal agreement. Importantly, most misclassifications occurred between adjacent categories rather than between extreme loading bins. This pattern implies that the wearable signal retains useful information about relative loading level even when exact threshold discrimination is imperfect. From a practical perspective, this is relevant because osteogenic exercise prescription is often based on broad loading ranges rather than exact point estimates. Accordingly, the present data suggest that wrist accelerometry may be more suitable for determining whether a jump is broadly low, moderate, or high in impact intensity than for quantifying the exact mechanical dose of an individual trial.

These findings should, however, be interpreted within the specific experimental context. The results apply only to maximal countermovement jumps performed with hands on hips under laboratory conditions. Constraining arm movement was necessary to reduce upper-limb contribution to the wrist signal and to standardize the task, but it also limits direct generalization to free-living impact activities or exercise tasks involving arm swing. In such settings, wrist acceleration may reflect upper-limb motion that is not tightly coupled to lower-limb loading. Therefore, the present study should be viewed as a validation under constrained conditions rather than as evidence that wrist-based monitoring can yet be transferred directly to all real-world movement patterns.

From a clinical and translational perspective, the most plausible application of this approach is session-level monitoring and conservative progression in home- or community-based osteogenic exercise programs. In practice, clinicians rarely need laboratory-grade quantification of the force produced in each jump; rather, they need a feasible way to determine whether exercise is being performed within a target loading range and whether progression is occurring over time. In this context, moderate ordinal agreement may be sufficient, particularly if signals are interpreted conservatively and aggregated across several jumps rather than relying on isolated trials. Multi-trial summaries, such as the median of the top-performing jumps within a session, may reduce instability near decision boundaries and better reflect the overall loading stimulus achieved during training. Thus, even though the method lacks precision for individual-level force estimation, it may still support pragmatic monitoring of whether participants are achieving loading targets in a broadly appropriate range.

The present results also help refine the interpretation of wearable-based monitoring in osteoporosis-preventive exercise. Earlier formulations of this work could suggest that wrist accelerometry acts as a relatively accurate proxy for force-platform measurement; the revised analyses support a more cautious conclusion. Wrist accelerometry appears to capture relative variation in impact intensity reasonably well, but its precision is insufficient for applications requiring exact per-jump quantification. Its utility is therefore better framed in terms of supporting broad categorization, adherence monitoring, and conservative progression frameworks rather than replacing force platforms or directly quantifying skeletal loading. This distinction is important because osteogenic adaptation depends not only on impact magnitude, but also on strain distribution, loading rate, and muscle-generated forces, none of which are directly resolved by a peripheral wrist sensor.

Several limitations should be acknowledged. First, although the revised analyses addressed within-subject dependence using participant-clustered inference and leave-one-subject-out validation, the findings have not yet been externally validated in an independent sample. Second, jump windowing remained manual and signal preprocessing was intentionally conservative, with no filtering or gravity removal, which may have increased trial-level noise. Third, the criterion was platform-derived vertical acceleration rather than direct skeletal strain or joint-specific loading, so conclusions should remain focused on pragmatic loading classification rather than exact bone-level mechanics. Fourth, the residual-based outlier filter was exploratory and was therefore treated as a sensitivity analysis rather than a primary result. Finally, the current data are restricted to maximal CMJs and do not establish whether similar performance would be observed for moderate-impact tasks, mixed movement patterns, or repeated exercise performed outside supervised laboratory environments.

Future studies should extend this work in several directions. External validation in independent cohorts is needed before broader application can be recommended. Automated alignment of wearable and force signals, together with standardized preprocessing pipelines, may improve robustness and reduce observer dependence. It will also be important to determine whether simple calibration models such as the present reduced model remain stable across devices, populations, and exercise modalities. At the application level, approaches such as threshold margins, multi-trial voting, or trend-based monitoring may convert noisy per-trial signals into safer and more interpretable feedback. Ultimately, longitudinal studies will be required to establish whether wearable-monitored impact programs translate into meaningful improvements in bone health and fracture prevention across diverse populations [27, 28].

## Conclusion

Wrist-worn accelerometry provided limited precision for individual-jump estimation of platform-derived loading during maximal countermovement jumps in postmenopausal women, even under constrained laboratory conditions. However, when combined with body mass, it retained moderate utility for coarse, bin-based classification of impact intensity and for capturing broad differences in loading level across trials.

Accordingly, the most defensible application of this approach is session-level monitoring and conservative progression in home- or community-based osteogenic exercise programs, rather than replacement of laboratory force-platform measurement. Wrist accelerometry may help identify whether impact loading falls broadly within low, moderate, or high osteogenic ranges, especially when multiple jumps are aggregated, but it should not be used as a precise surrogate for per-jump mechanical quantification.

Further work should externally validate these findings, test automated preprocessing and alignment procedures, and determine whether wearable-based classification of impact loading translates into meaningful improvements in bone health over time.

## Supplementary Information

Below is the link to the electronic supplementary material.

ESM 1 (PDF 524 KB)

## Data Availability

The datasets supporting the findings of this cross-sectional analysis are part of an ongoing randomized controlled trial (NCT06741956). Data will be made publicly available upon trial completion through the institutional repository of the University of Seville (IDUS) https://idus.us.es/home.
