# Supplementary material for: Quantifying impact loading for osteoporosis prevention: a study on the relationship between ground reaction forces and wearable impact data
Source: Arch Osteoporos. 2026 Sep 3;21(1):138. doi: 10.1007/s11657-026-01715-8 (PMC13541840; doi:10.1007/s11657-026-01715-8)
Supplement: Supplementary file 1 [file 11657_2026_1715_MOESM1_ESM.pdf]

Supplementary Material

Supplementary Table S1. Sensitivity analysis comparing the reduced and full anthropometric models using leave-one-subject-out cross-validation.

Dataset	Model	n_obs	n_subjects	R^2_LOSO	MAE_LOSO	RMSE_LOSO	Bias_LOSO	LoA_low_LOSO	LoA_high_LOSO	Accuracy_4class	κ_quadratic	Class-index MAE
main_unfiltered	Reduced (Weight + wrist VM)	125	37	0.4428	5.8287	7.9858	0.0510	-15.6639	15.7659	0.5440	0.6388	0.4880
sensitivity_iqr_filtered	Reduced (Weight + wrist VM)	121	36	0.5263	5.0790	6.5803	0.0952	-12.8544	13.0448	0.5950	0.6736	0.4215
main_unfiltered	Full anthropometric model	125	37	0.3853	6.2933	8.3877	0.0181	-16.4879	16.5241	0.4800	0.5989	0.5520
sensitivity_iqr_filtered	Full anthropometric model	121	36	0.4958	5.3410	6.7889	0.1529	-13.2053	13.5112	0.5289	0.6385	0.4876

Supplementary Table S2. Clustered regression coefficients for the full anthropometric model in the unfiltered and filtered datasets.

Panel A. Full anthropometric model, unfiltered dataset (125 trials from 37 participants)

**Predictor**	**β**	**95% CI**	**p-value**	**Standardized β**
Intercept	−12.264	−84.447 to 59.918	0.739	—
Body mass	−0.249	−0.601 to 0.104	0.167	−0.404
Height	0.124	−0.276 to 0.525	0.542	0.053
Waist circumference	0.161	−0.140 to 0.462	0.293	0.208
Hip circumference	−0.082	−0.611 to 0.447	0.762	−0.105
Wrist vector magnitude	0.772	0.543 to 1.000	<0.001	0.619

Panel B. Full anthropometric model, filtered sensitivity dataset (121 trials from 36 participants)

**Predictor**	**β**	**95% CI**	**p-value**	**Standardized β**
Intercept	−45.267	−91.484 to 0.950	0.055	—
Body mass	−0.395	−0.624 to −0.166	<0.001	−0.729
Height	0.276	−0.010 to 0.561	0.059	0.132
Waist circumference	0.159	−0.079 to 0.396	0.192	0.231
Hip circumference	0.110	−0.198 to 0.419	0.483	0.160
Wrist vector magnitude	0.720	0.487 to 0.953	<0.001	0.653

Note: Coefficients were estimated using ordinary least squares with participant-clustered robust standard errors. The full anthropometric model included body mass, height, waist circumference, hip circumference, and wrist vector magnitude. Standardized coefficients are shown to facilitate comparison of relative predictor importance across variables measured on different scales.

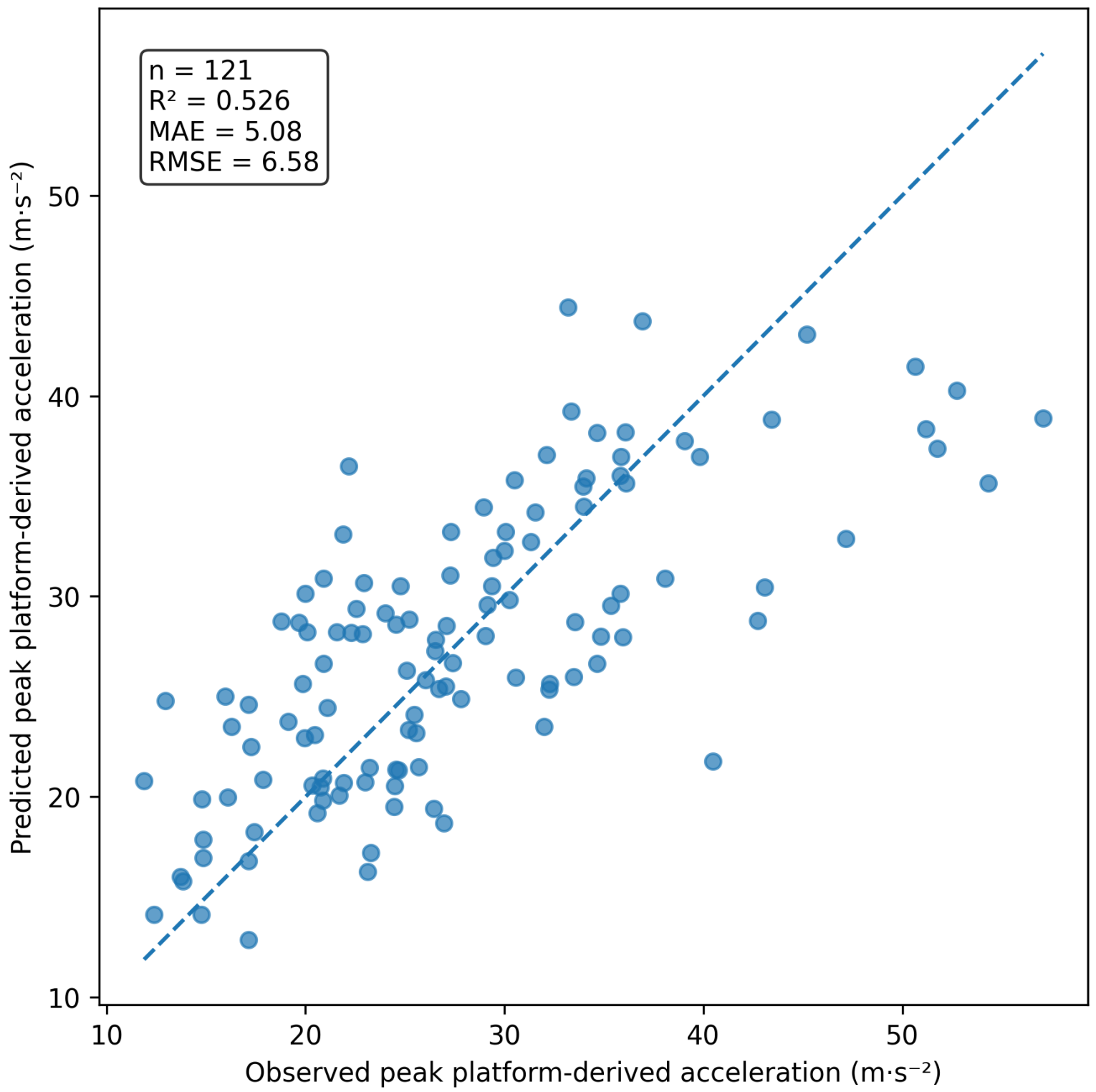

Supplementary Figure S1. Observed versus leave-one-subject-out predicted peak platform-derived vertical acceleration for the reduced model after residual-based outlier filtering. Scatter plot showing observed and cross-validated predicted values of peak platform-derived vertical acceleration ($m \cdot s^{-2}$) for the reduced model including wrist vector magnitude and body mass in the filtered sensitivity dataset (121 trials from 36 participants). Predictions were generated using leave-one-subject-out cross-validation. The dashed line indicates the line of identity.

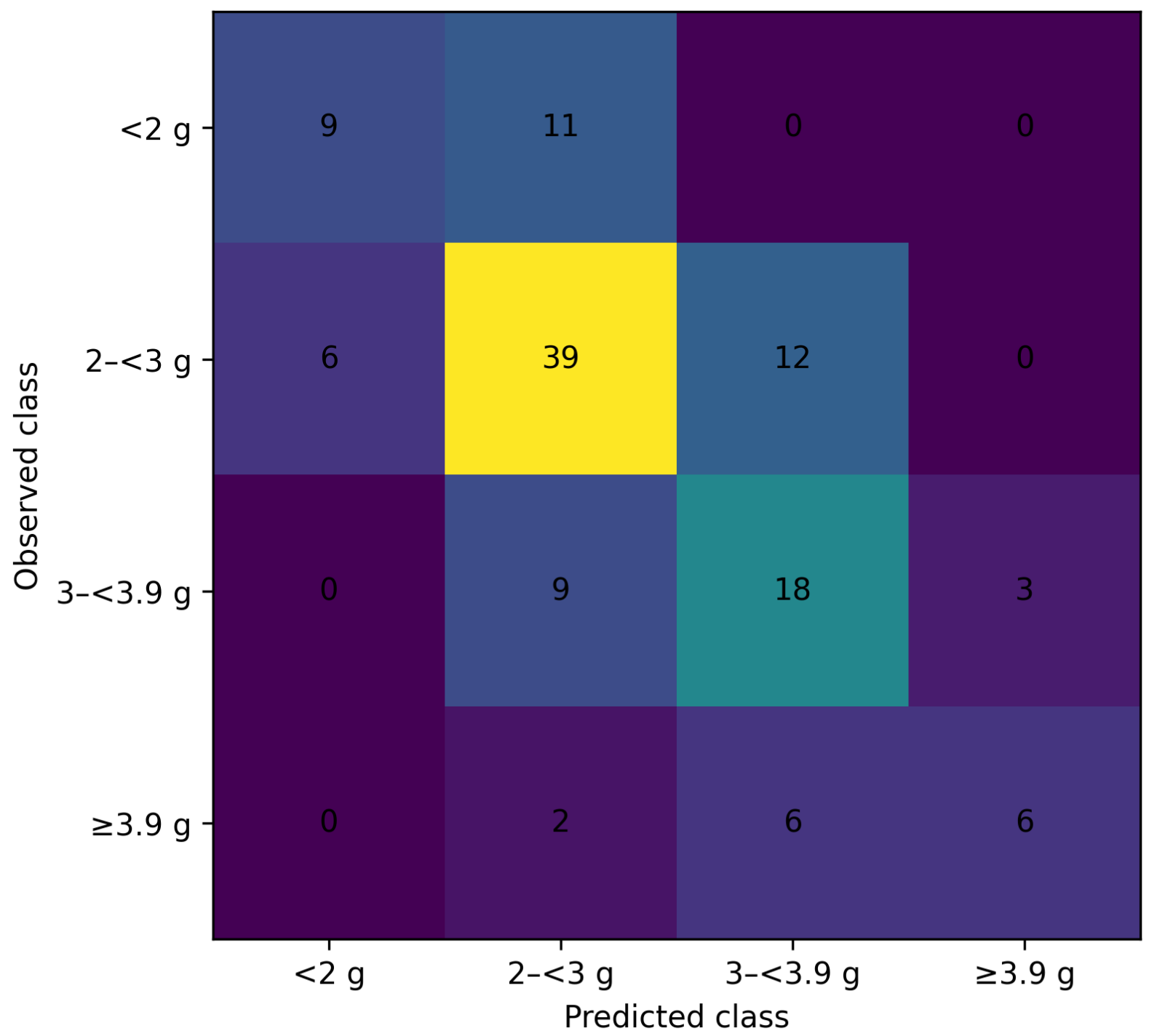

Supplementary Figure S2. Confusion matrix for four-class osteogenic intensity classification using leave-one-subject-out predictions from the reduced model after residual-based outlier filtering. Observed and predicted peak platform-derived vertical accelerations in the filtered sensitivity dataset were classified into four ordered osteogenic intensity categories using thresholds at 2 g, 3 g, and 3.9 g, corresponding to 19.62, 29.43, and 38.259 $m\cdot s^{-2}$, respectively. The resulting classes were <2 g, 2–<3 g, 3–<3.9 g, and ≥3.9 g. Values are shown as absolute counts. Classification performance improved modestly after filtering, but the overall interpretation remained unchanged.

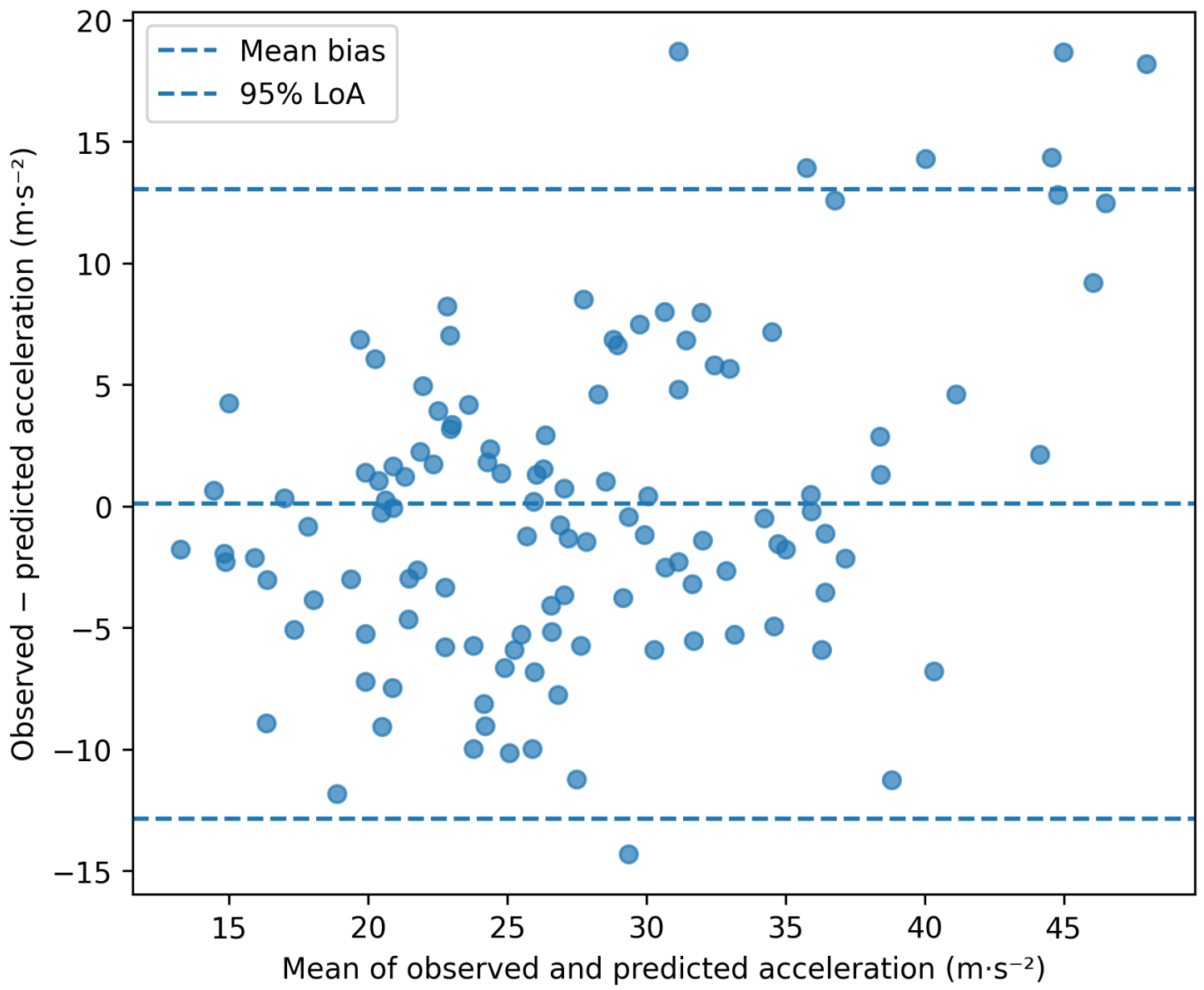

Supplementary Figure S3. Bland–Altman plot comparing observed and leave-one-subject-out predicted peak platform-derived vertical acceleration for the reduced model after residual-based outlier filtering. The x-axis shows the mean of observed and predicted peak platform-derived vertical acceleration ($m \cdot s^{-2}$), and the y-axis shows the difference between observed and predicted values. The solid horizontal line indicates the mean bias (0.10 $m \cdot s^{-2}$), and the dashed lines indicate the 95% limits of agreement (−12.85 to 13.04 $m \cdot s^{-2}$). Results are based on leave-one-subject-out predictions from the reduced model in the filtered sensitivity dataset (121 trials from 36 participants).
